# Epidemiology and socioeconomic correlates of colorectal cancer in Asia in 2020 and its projection to 2040

**DOI:** 10.1038/s41598-025-12545-y

**Published:** 2025-07-22

**Authors:** Seyed Ehsan Mousavi, Mehran Ilaghi, Romina Hamidi Rad, Seyed Aria Nejadghaderi

**Affiliations:** 1 Neurosciences Research Center, Aging Research Institute, Tabriz University of Medical Sciences, Tabriz, Iran; 2 Department of Community Medicine, Social Determinants of Health Research Center, Faculty of Medicine, Tabriz University of Medical Sciences, Tabriz, Iran; 3 Institute of Neuropharmacology, Kerman Neuroscience Research Center, Kerman University of Medical Sciences, Kerman, Iran; 4 Department of Medicine, Tehran Medical Branch, Islamic Azad University, Tehran, Iran; 5 HIV/STI Surveillance Research Center, and WHO Collaborating Center for HIV Surveillance, Institute for Futures Studies in Health, Kerman University of Medical Sciences, Kerman, Iran; 6 Knowledge Hub for Migrant and Refugee Health, Institute for Futures Studies in Health, Kerman University of Medical Sciences, Kerman, Iran

**Keywords:** Colorectal neoplasm, Colon cancer, Epidemiology, Prevalence, Incidence, Mortality, GLOBOCAN, Colorectal cancer, Epidemiology

## Abstract

Asia bears a disproportionate and rapidly rising burden of colorectal cancer (CRC). However, the incidence and mortality trends vary significantly between Asian countries, mainly due to the diversity of socioeconomic factors and the implementation of screening programs. This study aimed to report the contemporary distribution, socioeconomic correlates, and projections for future trends of CRC across Asia. The Global Cancer Observatory (GLOBOCAN) for the year 2020 was used to obtain data on prevalence, incidence, and mortality rates of CRC. We calculated mortality-to-incidence ratios (MIRs), age-standardized incidence and mortality rates (ASIR and ASMR), crude rates, numbers, and 5-year prevalent cases and rates by age, sex, and subregions of Asia. We assessed the correlation between indicators and human development index (HDI) and the ratio of current health expenditure (CHE) to gross domestic product (GDP) using Pearson’s correlation coefficient. Estimated incidence or mortality rates between 2025 and 2040 were calculated by multiplying age-specific rates for 2020 by the estimated population between 2025 and 2040. In Asia, the 5-year prevalence rate, ASIR, and ASMR of CRC were 55.60, 17.30, and 8.40 per 100,000, respectively. The highest crude incidence and mortality rates were in the 70 + age group. Males had higher ASIRs than females (20.80 vs. 14.00 per 100,000) in Asia. MIRs for men and women were 0.49 globally and 0.50 and 0.51 in Asia, respectively. A positive significant correlation was observed between HDI and both the ASIR and ASMR. A strong negative correlation was observed between HDI and MIR. The number of incident and mortality cases are estimated to increase by 71.10% and 85.10% in 2040, respectively. CRC is a significant public health concern in Asia, with substantially high incidence and mortality rates in East Asia and lower quality of care and survival in less developed regions of the continent. Resource allocation prioritizing population-based screenings alongside capacity building around specialized care centers is crucial across the Asian countries.

## Introduction

Colorectal cancer (CRC), cancers of the colon and rectum combined, is the third most commonly diagnosed cancer and the second leading cause of cancer deaths worldwide^1,2^. In 2020, there were approximately 1.9 million newly reported cases of CRC, with more than 900,000 deaths attributed to CRC globally^3^.

Asia, which harbors over half of the world’s population, has witnessed a rapid increase in the incidence and mortality burdens of CRC in recent decades^4^. According to estimates, Asia has the highest overall cancer burden compared to the other regions in the world^5^. Based on the global cancer observatory (GLOBOCAN) estimates, almost one million new cases of CRC were diagnosed in Asia in 2020, accounting for 10.6% of all cancers in the continent. Moreover, CRC also resulted in approximately 500,000 deaths in Asia in 2020, making it the fourth most common cause of cancer-related deaths in Asia^5^.

Although CRC was believed to be infrequent in Asia more than three decades ago, contrasting with higher incidences in North America and Europe^6^, lifestyle risk factors, including increased intake of red and processed meat, elevated alcohol consumption, cigarette smoking, obesity, diabetes, in addition to improvements in screening programs and diagnostic methods have been associated with changing trends^7,8^.

Among the Asian countries, several countries, including South Korea, Japan, and Singapore where CRC is highly prevalent, have implemented population-based screening programs^4,9^. Nonetheless, the majority of countries currently lack any structured CRC screening initiatives, with only a limited number implementing pilot or opportunistic screening practices. Furthermore, many Asian regions continue to face challenges in terms of clinical capacity, characterized by insufficient infrastructure, such as endoscopy units and cancer registries, along with limitations in human resources for the screening and management of lesions detected through screening processes. Accordingly, socioeconomic disparities, including poverty and lack of insurance, impedes individual’s access to CRC screening, care, and subsequent management^10–12^. In conjunction with other existing challenges, a notable deficiency persists in the formulation of national guidelines and an auditing system for CRC screening throughout Asian regions^13^.

Although the global epidemiology of CRC has been studied in recent studies, no study has so far assessed the CRC incidence and mortality and their socioeconomic correlates across Asia based on the updated GLOBOCAN 2020 database. Moreover, an estimation of CRC projection in upcoming decades seems to be crucial considering the high burden of CRC across the continent. Therefore, in this study, we aimed to comprehensively analyze the contemporary prevalence, incidence, and mortality of CRC across all countries in Asia using the latest GLOBOCAN 2020 data. Moreover, we estimated projections of CRC incidence and mortality cases up to 2040. Additionally, we evaluated the correlations of sociodemographic factors with CRC epidemiological indicators across Asian countries to provide an updated understanding of the CRC burden and its socioeconomic determinants, facilitating the formation of cancer control policies in the region.

## Methods

### Study design and data sources

GLOBOCAN is a public access online database established by the International Agency for Research on Cancer and World Health Organization (WHO) to provide an estimate of the global cancer burden for different types of cancer^14^. We retrieved age-, sex-, and country-specific data for CRC. It was defined as the International Classification of Disease version 10 codes of C18-20.

We extracted the 2020 population data from the United Nations’ 2019 World Population revision. We also extracted human development index (HDI) rates from the United Nations Development Programme Human Development Report Office^15^. We extracted data to estimate the percentage of current health expenditure to gross domestic product ratio (CHE/GDP%) for 2019 from WHO’s Global Health Observatory^16^. In designing and reporting the results of this study, we followed the Guidelines for Accurate and Transparent Health Estimates Reporting^17^ as well as the Strengthening the Reporting of Observational Studies in Epidemiology^18^.

### Variables

This study discussed the epidemiology of CRC in Asia, classified by sex, age, and country. Three epidemiological measures of CRC, including prevalence, incidence, and mortality, were extracted. Mortality-to-incidence ratios (MIRs) were calculated. Cumulative risk percentages of incidence and mortality were also used to describe the risk of developing colon cancer and mortality from it in people under 75 years of age.

#### Incidence

Different methods were used to calculate incidence rates for specific age and sex demographics, summarized in descending order of importance. National incidence rates up to 2020 were projected. The most recently observed incidence rates at either national or regional levels were applied to the 2020 population. Estimations were obtained from modeling methods, using MIRs from cancer registries within particular countries, neighboring regions, or survival estimation. In the absence of data, rates from neighboring countries or local registries were used^19^.

#### Mortality

Methods to obtain age- and sex-specific mortality rates were largely similar to those of obtaining incidence rates described earlier: projecting observed national mortality rates to 2020, applying rates to the 2020 population, estimating rates using modelling and MIR reported from cancer registries of neighboring countries, and averaging the rates of the of neighboring countries selected^19^.

#### Prevalence

Prevalence estimates for different age, sex, and regional groups for 2020 were calculated using ratios of incidence to prevalence from Nordic countries for the period 2006–2015. Some exceptions were excluded from scaling with HDI index: cancers of the breast, prostate, esophagus, stomach, liver, gallbladder, pancreas, lung, mesothelioma, and cancer of the stomach and liver from South Korea and Japan^19^. The following formula was used for the calculation of the prevalence in each country:

$${Prevalenc}_{Country}= {Incidence}_{Country}\times \frac{{Prevalenc}_{Nordic}}{{Incidence}_{Nordic}} \times \frac{{HDI}_{Country}}{{HDI}_{Nordic}}$$

#### MIR

The MIR is defined as the crude mortality rate over the crude incidence rate and indicates of the quality of healthcare, with a low value indicating better cancer care in terms of screening, treatment, and overall disease management^20^.

#### CHE/GDP%

This ratio, CHE/GDP%, highlights the importance of the health sector to the overall economy as it measures the financial resources allocated to the health sector. In the data from 2021, the values of this index ranged from 5.49 to 8.70 based on WHO regions^16^.

#### HDI

The HDI is an indicator based on life expectancy at birth, education, the projected length of educational years for children within the school-going age, and income per capita in United States dollars for the purpose of providing a socioeconomic status. In this study, HDI is used to investigate the cancer burden in countries with different levels of development. Countries fall into four levels according to their HDI—low (below 0.555), medium (0.555 to 0.699), high (0.700 to 0.799), and very high (0.800 and above)^15^.

### Statistical analysis

Crude incidence and mortality rates, number of new cases and mortalities, age-standardized incidence and mortality rate (ASIR and ASMR), 5-year prevalent cases and prevalence rate were presented in the forms of tables and figures per 100,000 population. We calculated age-standardized rates using the direct standardization method on the 1966 Segi-Doll World standard population and categorized age groups at intervals (0–9, 10–19, 20–29, 30–39, 40–49, 50–59, 60–69, and 70+). We analyzed the association between incidence and mortality rates and estimated MIR, HDI, and CHE/GDP% among countries with available data using a bivariate correlation test and reported with 95% confidence intervals (CIs) and two-sided p-values. The results were reported using Pearson’s correlation coefficient based on three categories of absolute values: strong (> 0.5), moderate (0.5–0.3), and weak (< 0.3). A P-value of less than 0.5 from a two-sided test was regarded as statistically significant. We calculated the estimated numbers of incidence and mortality between 2025 and 2040 by multiplying age-specific rates calculated for 2020 by the estimated population for the years 2025 to 2040:

$$\text{Estimated incidence or mortality }\left(2025\text{ to }2040\right)=\text{age specific rates for }2020\times \text{estimated population }(2025\text{ to }2040)$$

All data cleaning, analysis, and visualization were done using R statistical software, version 4.3.2^21^.

## Results

### Prevalence, incidence, mortality, and MIR among both sexes

**Fig. 1:**
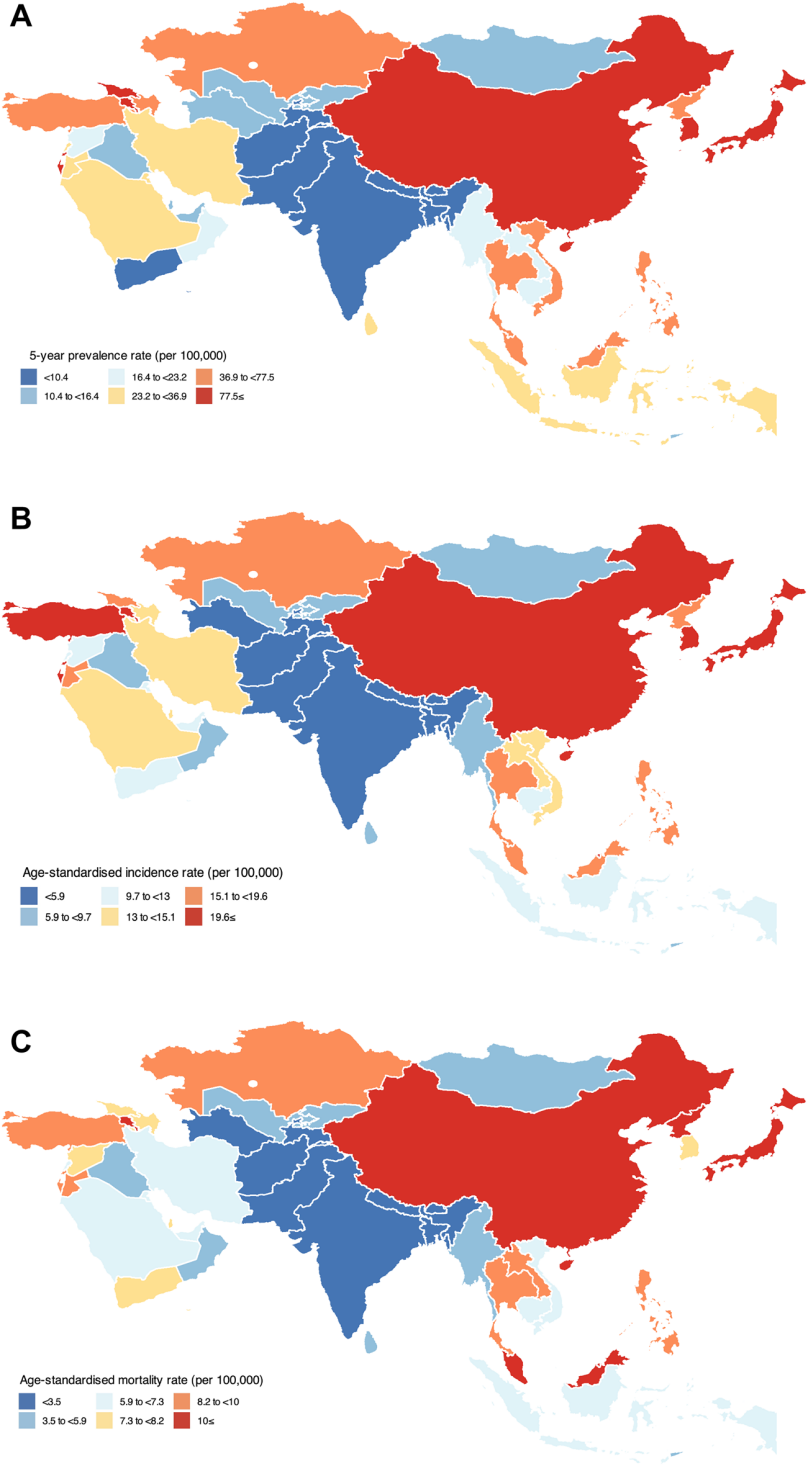
Distribution of (**A**) five-year prevalence rate, (**B**) age-standardized incidence and (**C**) age-standardized mortality rates per 100,000 of colorectal cancer among both sexes in Asia in 2020.

With a 5-year prevalence rate of 65.60, there were 5,111,957 cases of 5-year prevalence globally, including 2,581,300 cases in Asia, with a 5-year prevalence rate of 55.60 in Asia. The continent of Europe had the highest 5-year prevalence rate at 199.70. With a 119.90 5-year prevalence rate, Eastern Asia had the highest 5-year prevalence rate among Asian subregions, while the lowest rates were 10.40 in South-Central Asia (Table 1). With 359.20 per 100,000 population, Japan had the highest 5-year prevalence rate in Asian countries; followed by 186.50 in Singapore and 168.50 in the Republic of Korea (Fig. 1A and Supplementary File 1).

**Table 1:** Colorectal cancer five-year prevalence, incidence, and mortality metrics in 2020 for different geographic location in both sexes, males, and females.

Location	Prevalence		Incidence				Mortality				**MIR**
	5-year prevalent cases	5-year prevalence rate	Number	Crude rate	ASR	Cumulative risk (%)	Number	Crude rate	ASR	Cumulative risk (%)	
**Both sexes**											
World	5,111,957	65.6	1,880,725	24.1	19	5.37	915,880	11.7	8.8	3.22	0.49
Asia	2,581,300	55.6	992,755	21.4	17.3	4.89	498,329	10.7	8.4	3.16	0.5
Eastern Asia	2,011,339	119.9	751,457	44.8	25.6	7.06	364,976	21.7	11.7	4.41	0.49
South-Central Asia	210,533	10.4	95,656	4.7	5.1	1.21	55,511	2.8	3	0.82	0.6
South-Eastern Asia	253,319	37.9	104,714	15.7	14.5	3.85	56,009	8.4	7.7	2.73	0.53
Western Asia	106,109	38.1	40,928	14.7	16.5	4.22	21,833	7.8	8.7	2.84	0.53
Continents											
Africa	118,716	8.9	60,078	4.5	7.6	1.91	38,814	2.9	5.1	1.54	0.65
Europe	1,495,342	199.7	507,044	67.7	29.5	7.79	240,797	32.2	12.1	4.25	0.48
Latin America and the Caribbean	331,319	50.7	129,596	19.8	16	4.47	68,059	10.4	8.1	2.73	0.53
Northern America	524,289	142.1	171,398	46.5	24.8	5.81	62,465	16.9	8	2.51	0.36
Oceania	60,991	142.9	19,854	46.5	28.5	8.06	7416	17.4	9	3.83	0.37
Males											
World	2,794,971	71.1	1,044,254	26.6	22.9	6.49	506,221	12.9	10.8	3.94	0.49
Asia	1,451,555	61.2	567,710	23.9	20.8	5.83	283,975	12	10.4	3.79	0.5
Eastern Asia	1,129,827	132.1	428,350	50.1	31	8.48	206,448	24.1	14.6	5.36	0.48
South-Central Asia	122,246	11.8	56,943	5.5	6.1	1.52	33,712	3.2	3.7	1.04	0.58
South-Eastern Asia	140,343	42	59,278	17.8	18	4.89	31,593	9.5	9.9	3.52	0.53
Western Asia	59,139	40.5	23,139	15.9	19.6	5.24	12,222	8.4	10.6	3.54	0.53
Continents											
Africa	59,535	8.9	30,899	4.6	8.5	2.22	19,922	3	5.7	1.81	0.65
Europe	809,077	223.7	277,387	76.7	37.3	10.1	130,285	36	15.8	5.65	0.47
Latin America and the Caribbean	164,671	51.2	65,403	20.3	18	5.01	34,469	10.7	9.3	3.11	0.53
Northern America	277,523	152	92,093	50.4	28.4	6.71	33,557	18.4	9.6	2.93	0.36
Oceania	32,610	152.6	10,762	50.4	32.8	9.16	4013	18.8	10.9	4.35	0.37
Females											
World	2,316,986	60	836,471	21.6	15.6	4.5	409,659	10.6	7	2.68	0.49
Asia	1,129,745	49.8	425,045	18.7	14	4.11	214,354	9.5	6.7	2.66	0.51
Eastern Asia	881,512	107.2	323,107	39.3	20.7	5.9	158,528	19.3	9.1	3.68	0.49
South-Central Asia	88,287	9	38,713	4	4.1	0.93	21,799	2.2	2.3	0.62	0.55
South-Eastern Asia	112,976	33.8	45,436	13.6	11.6	3.11	24,416	7.3	6	2.2	0.54
Western Asia	46,970	35.4	17,789	13.4	13.8	3.46	9611	7.2	7.2	2.34	0.54
Continents											
Africa	59,181	8.8	29,179	4.4	6.9	1.68	18,892	2.8	4.6	1.35	0.64
Europe	686,265	177.2	229,657	59.3	23.5	6.21	110,512	28.5	9.3	3.34	0.48
Latin America and the Caribbean	166,648	50.1	64,193	19.3	14.3	4.05	33,590	10.1	7.1	2.44	0.52
Northern America	246,766	132.5	79,305	42.6	21.6	5.06	28,908	15.5	6.6	2.17	0.36
Oceania	28,381	133.2	9092	42.7	24.5	7.09	3403	16	7.2	3.37	0.37

*ASR* Age-standardized rate, *MIR* Mortality-to-incidence ratio. Rates are presented per 100,000 population.

A total of 1,880,725 new cases of CRC was observed in 2020 globally, with a cumulative risk of 5.37%, a crude rate of 24.10, and an ASIR of 19.00 per 100,000 population. The continents of Europe and Oceania showed the highest ASIRs at 29.50 and 28.50, respectively. Asia showed 992,755 new cases, with a crude rate of 21.40 and an ASIR of 17.30 per 100,000. Eastern Asia accounted for the highest number of cases among Asian regions, with 751,457 new cases. Cumulative risk, crude rate, and ASIR for Eastern Asia were 7.06%, 44.80, and 25.60, respectively. The lowest ASIR was in South-Central Asia (5.1 per 100,000) (Table 1). Japan (38.20), Brunei Darussalam (34.90), and Singapore (32.40) had the highest ASIRs in Asia (Fig. 1B and Supplementary File 1).

There were 915,880 mortalities globally, with a cumulative risk of 3.22%, a crude rate of 11.70, an ASMR of 8.8 per 100,000, and an MIR of 0.49. By continent, Europe had the highest ASMR at 12.50. Among Asian subregions, Eastern Asia accounted for the highest crude rate (21.70) and ASMR (11.70). The MIR was highest in South-Central Asia (0.60), followed by South-Eastern Asia (0.53) and Western Asia (0.53) (Table 1). Singapore (16.10), Brunei Darussalam (14.60), and China (11.90) had the highest ASMRs (Fig. 1C and Supplementary File 1).

### Epidemiology by age

**Fig. 2:**
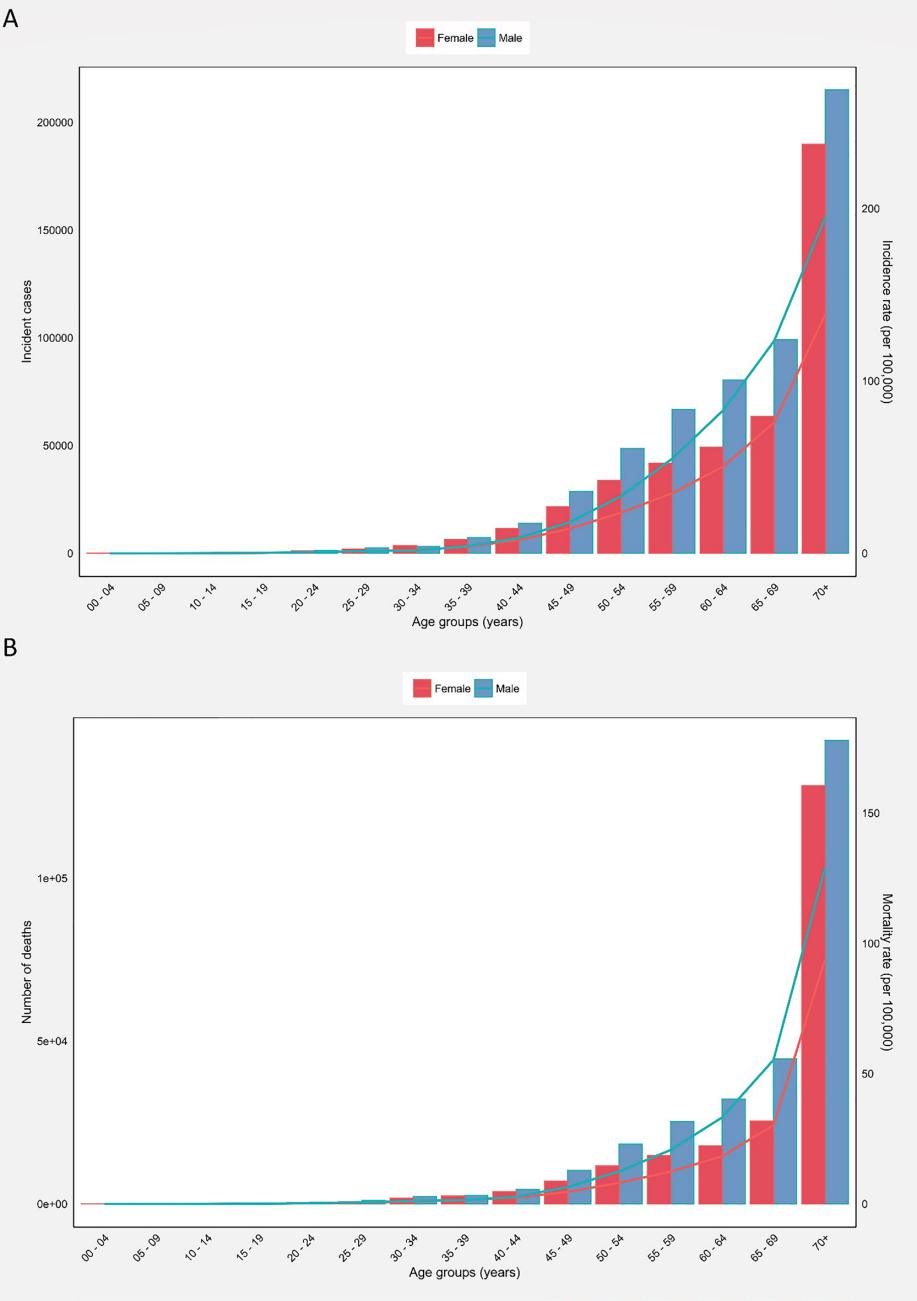
(**A**) Incident numbers and incidence rate, and (**B**) mortality numbers and mortality rate of colorectal cancer among males and females in each age group in Asia in 2020.

For both incidence and mortality, rates increased progressively across each successive five-year age group, with a particularly marked acceleration after age 50. In addition, the number of incident cases and deaths increased with ageing. Males had higher incident and death cases and rates in almost all age groups (Fig. 2A,B). The highest number of new cases and rates were in the age group of 70 years and older (404,852 cases, crude rate of 163.70, and cumulative risk of 3.59%), while the lowest incidence was in 0- to 9-year-olds (105 cases and crude rate 0.01). The crude incidence rate in 70 years and older was 194.90 in men compared to 138.60 in women. There were 29 mortalities in the age group 0 to 9 years in Asia, accounting for the lowest ones in all age groups. The highest number of mortalities belonged to the 70 + age group (270,668 cases, crude rate of 109.50, cumulative risk of 2.65%, and MIR of 0.67). The highest MIRs for males and females were 0.66 and 0.68, respectively, which were both in the 70 + age group (Table 2).

**Table 2:** Colorectal cancer incidence, mortality, and mortality-to-incidence ratio metrics in Asia in 2020 for different age groups among both sexes, males, and females.

Age group	Incidence			Mortality			MIR
	Number	Crude rate	Cumulative risk (%)	Number	Crude rate	Cumulative risk (%)	
Both sexes							
0 to 9	105	0.01	0	29	0	0	0
10 to 19	1102	0.15	0	389	0.05	0	0.33
20 to 29	7017	0.98	0.01	2606	0.36	0	0.37
30 to 39	20,395	2.8	0.03	9055	1.3	0.01	0.46
40 to 49	75,865	12.3	0.12	25,434	4.1	0.04	0.33
50 to 59	191,029	35.9	0.37	70,144	13.2	0.13	0.37
60 to 69	292,390	81.4	0.82	120,004	33.4	0.34	0.41
70 +	404,852	163.7	3.59	270,668	109.5	2.65	0.67
Males							
0 to 9	65	0.02	0	17	0	0	0
10 to 19	635	0.17	0	248	0.07	0	0.41
20 to 29	3825	1	0.01	1561	0.42	0	0.42
30 to 39	10,472	2.8	0.03	4885	1.3	0.01	0.46
40 to 49	42,629	13.5	0.13	14,668	4.7	0.05	0.35
50 to 59	115,355	42.9	0.44	43,596	16.2	0.17	0.38
60 to 69	179,641	101.2	1.03	76,735	43.2	0.44	0.43
70 +	215,088	194.9	4.26	142,265	128.9	3.14	0.66
Females							
0 to 9	40	0.01	0	12	0	0	0
10 to 19	467	0.14	0	141	0.04	0	0.29
20 to 29	3192	0.93	0.01	1045	0.3	0	0.32
30 to 39	9923	2.8	0.03	4170	1.2	0.01	0.43
40 to 49	33,236	11	0.11	10,766	3.6	0.04	0.33
50 to 59	75,674	28.7	0.29	26,548	10.1	0.1	0.35
60 to 69	112,749	62.1	0.63	43,269	23.4	0.24	0.38
70+	189,764	138.6	3.07	128,403	93.8	2.25	0.68

*MIR* Mortality-to-incidence ratio.

### Epidemiology by sex

The 5-year prevalence rates for men and women were 71.10 and 60.00 globally and 61.20 and 49.80 in Asia, respectively. Also, 1,044,254 (55.51%) of the new cases globally were male, while 836,471 (44.49%) were female. The incident cases were 567,710 (57.18%) for males and 425,045 (42.82%) for females in Asia. MIRs in men and women were 0.50 and 0.51 in Asia, respectively (Table 1).

**Fig. 3:**
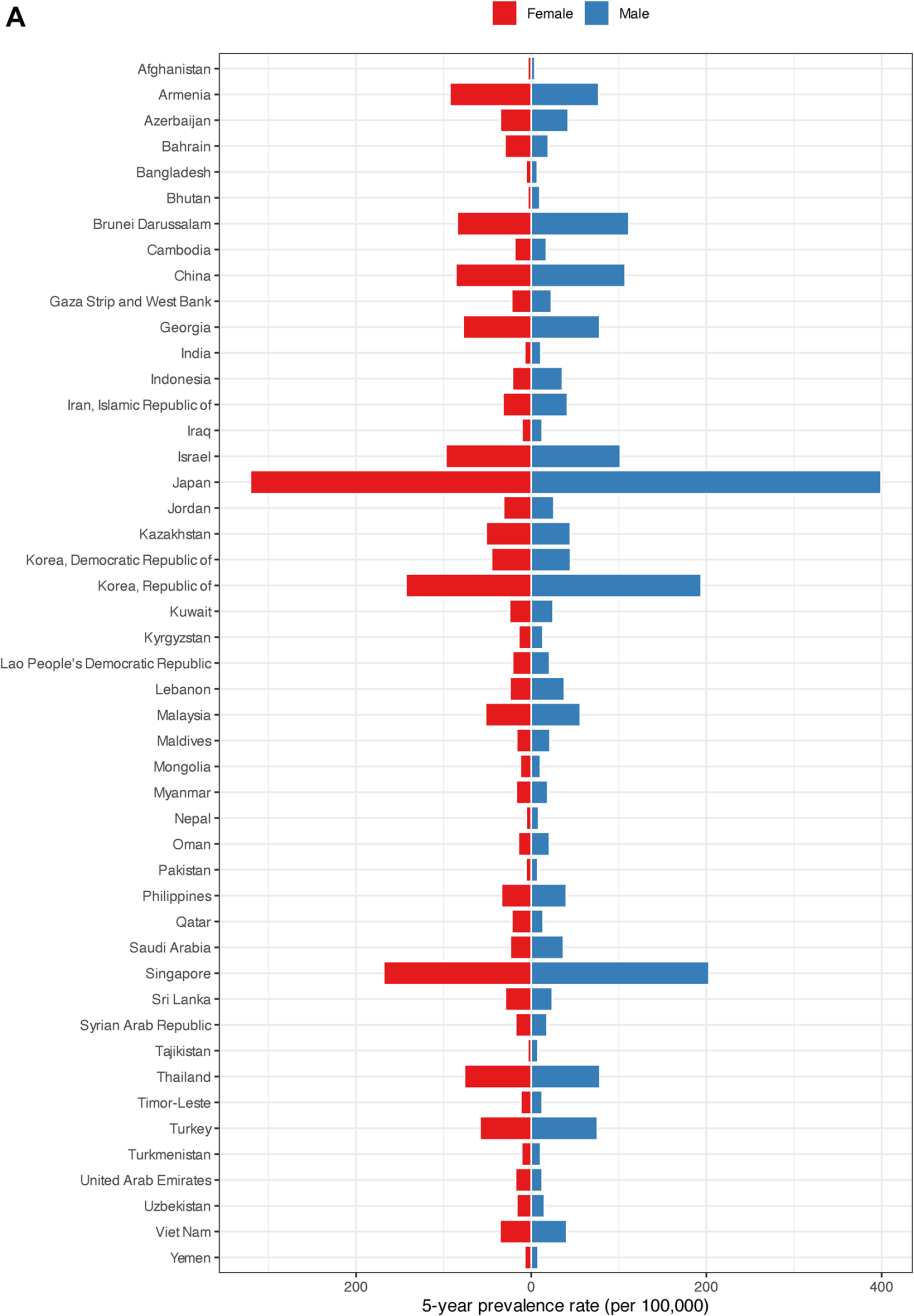

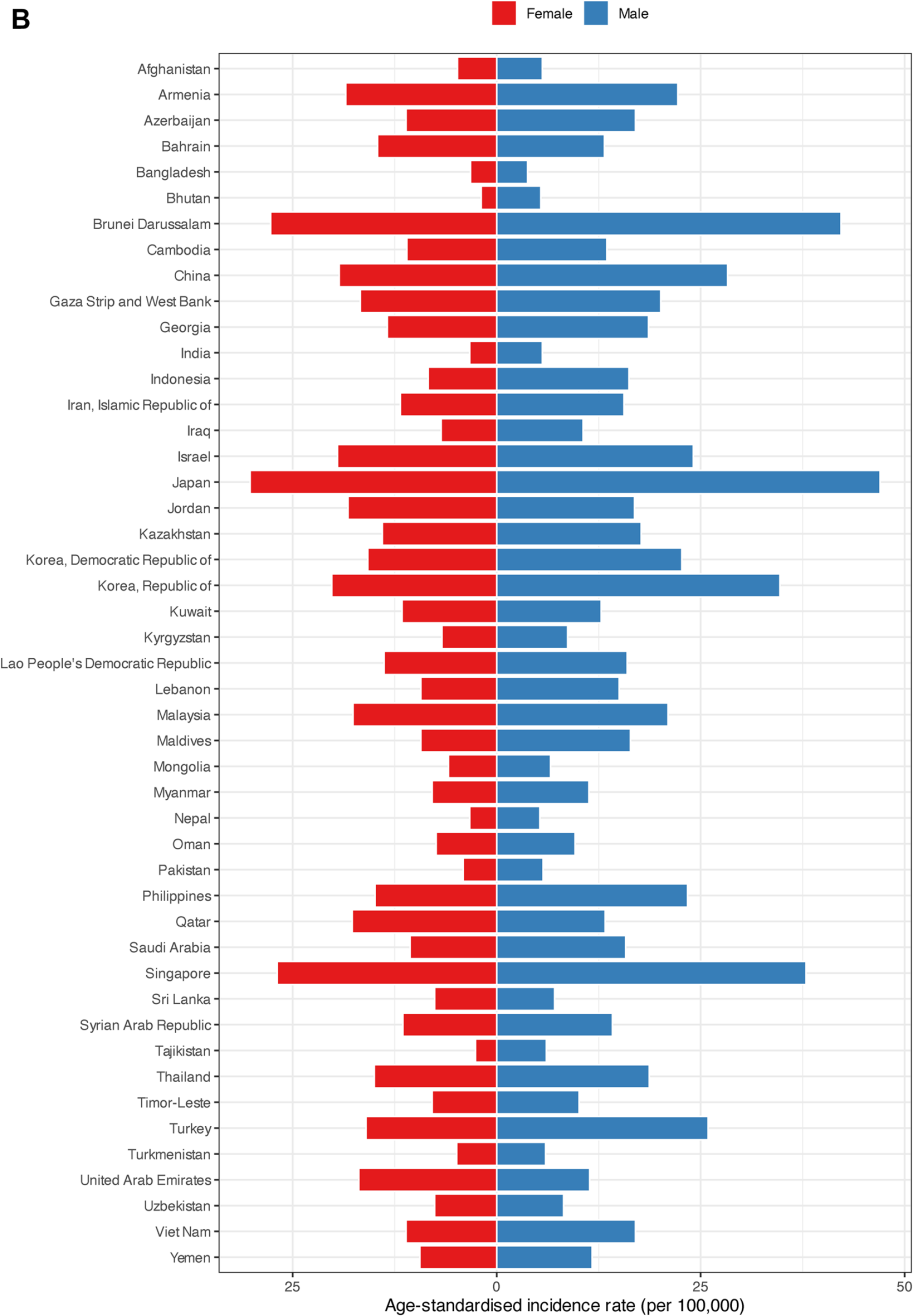

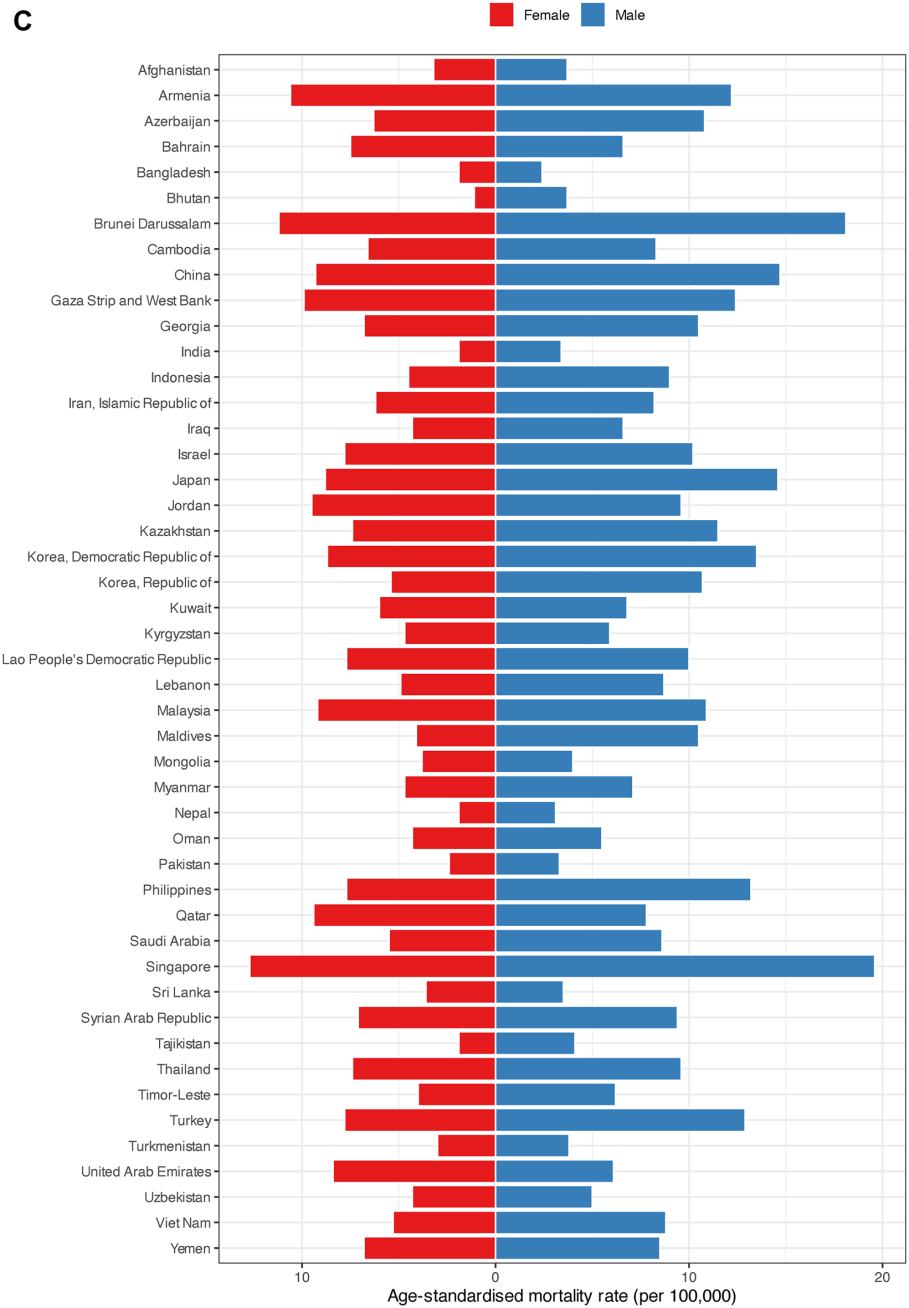
(**A**) Five-year prevalence rate, (**B**) age-standardized incidence and (**C**) age-standardized mortality rates per 100,000 of colorectal cancer in Asia in 2020, by country and sex.

The 5-year prevalence rates were highest among men in Japan (399.50), Singapore (202.90), and the Republic of Korea (193.90). Afghanistan had the lowest 5-year prevalence rate for men at 4.20. The highest 5-year prevalence rates for women were observed in Japan, Brunei Darussalam, and the Republic of Korea, recording rates of 320.60, 168.40, and 143.10, respectively. Bhutan reported the lowest 5-year prevalence rate for females at 3.90 (Fig. 3A, Figure S1, Figure S2, and Supplementary File 1).

Among men in Asia, the highest ASIRs were observed in Japan (47.00), Brunei Darussalam (42.20), and Singapore (37.90). Contrarily, Bangladesh showed the lowest ASIR for men at 3.80. For women, Japan (30.20), Brunei Darussalam (27.70), and Singapore (26.90) had the highest ASIRs in Asia. Bhutan had the lowest ASIR for females at 1.90 (Fig. 3B, Figs. S3, S4, and Supplementary File 1).

The highest ASMRs for men in Asia belonged to Singapore, Brunei Darussalam, and China at 19.60, 18.10, and 14.70, respectively. Bangladesh marked the lowest ASMR among men at 2.40. Singapore with 12.70, Brunei Darussalam with 11.20, and Armenia with 10.60 had the highest ASMRs for females. Bhutan had the lowest ASMR for women by 1.10 (Fig. 3C, Figs. S5, S6, and Supplementary File 1).

Yemen (0.69), Bhutan (0.67), and Kyrgyzstan (0.66) recorded the highest MIRs for men. Republic of Korea showed the lowest MIR for men at 0.33. Among females, Kyrgyzstan and Yemen had the highest MIRs at 0.71. Republic of Korea marked the lowest MIR for women in Asia at 0.36 (Supplementary File 1).

### Correlations between ASIR, ASMR, MIR, and HDI and CHE/GDP%

**Fig. 4:**
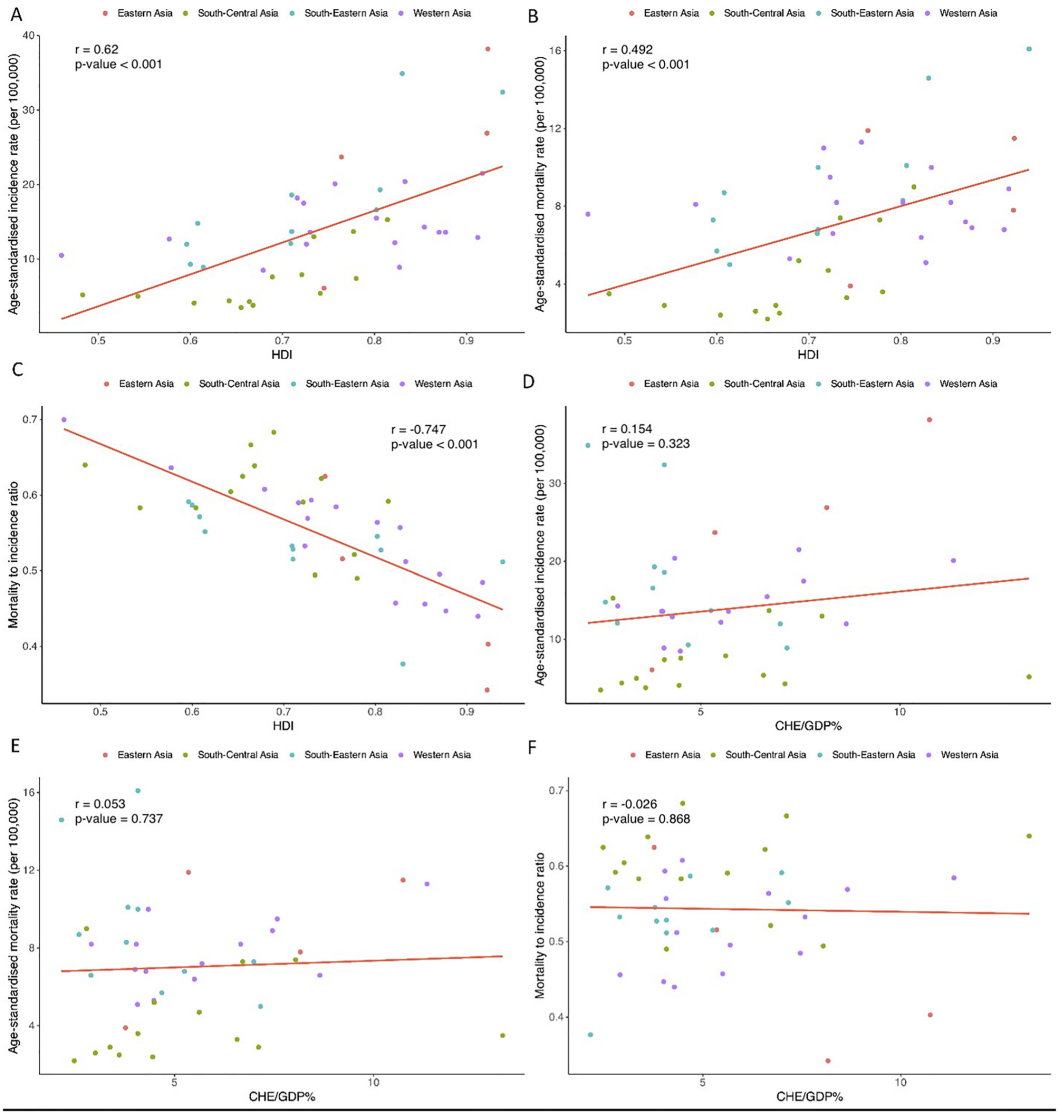
Correlation of human development index (HDI) with (**A**) age-standardized incidence rate, (**B**) age-standardized mortality rate, and (**C**) mortality-to-incidence ratio. Correlation of the current healthcare expenditure to gross domestic product (CHE/GDP%) with (**D**) age-standardized incidence rate, (**E**) age-standardized mortality rate, and (**F**) mortality-to-incidence ratio of colorectal cancer in Asia in 2020.

A significantly strong positive correlation was observed between HDI and ASIR (correlation coefficient: 0.620, 95% CI: 0.402, 0.771, p-value < 0.001) (Fig. 4A). There was also a moderate positive correlation between HDI and ASMR (correlation coefficient: 0.492, 95% CI: 0.236, 0.685, p-value < 0.001) (Fig. 4B). A strong negative correlation was observed between HDI and MIR (correlation coefficient: -0.747, 95% CI: -0.852, -0.583, p-value < 0.001) (Fig. 4C).

There were no significant correlations between health expenditure as CHE/GDP% and ASIR (Correlation coefficient: 0.154, p-value: 0.323) (Fig. 4D), ASMR (correlation coefficient: 0.053, p-value: 0.737) (Fig. 4E), and MIR (correlation coefficient: -0.026, p-value: 0.868) (Fig. 4F).

### Incidence and mortality projections in Asia

**Fig. 5:**
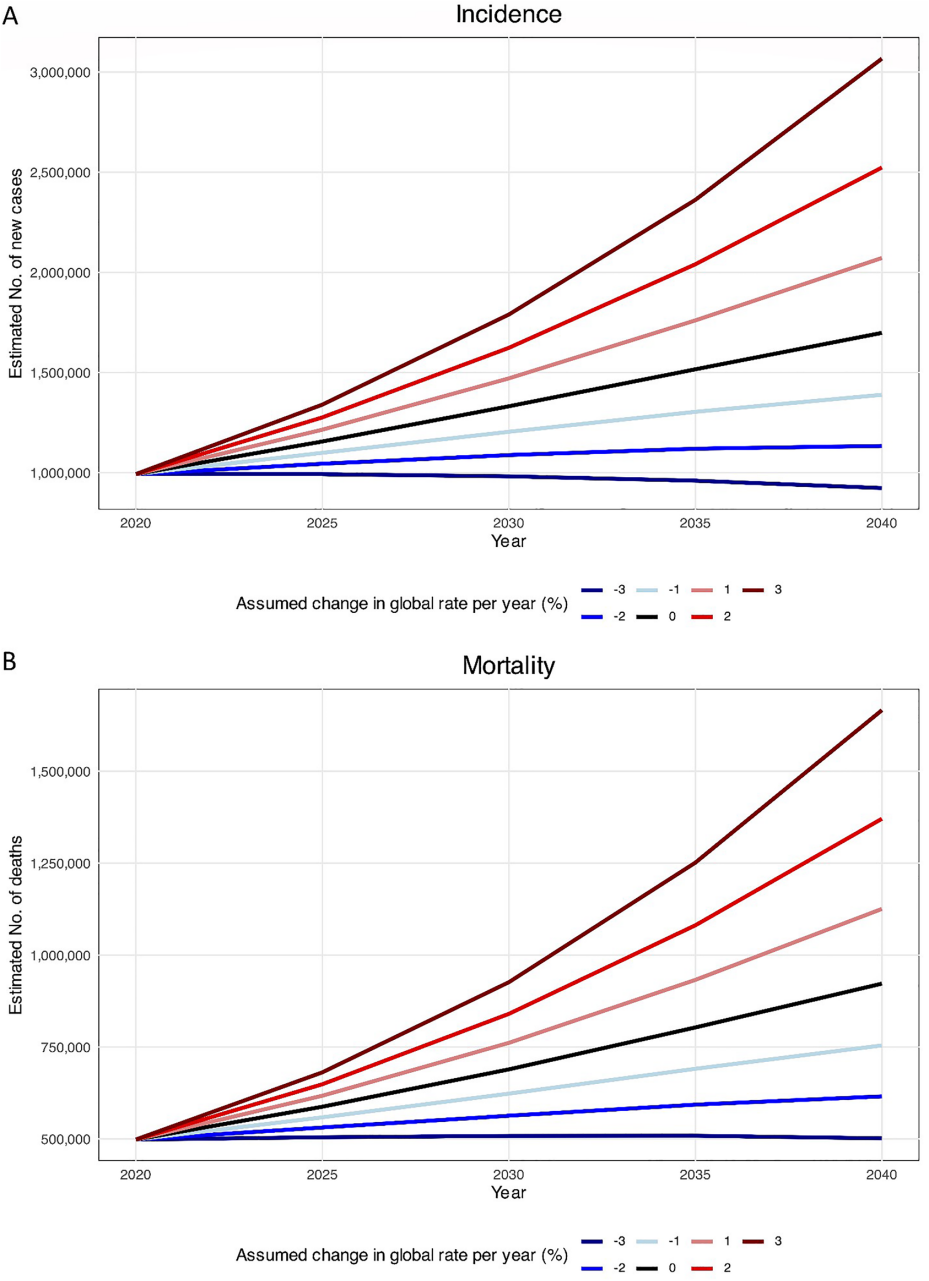
Estimated colorectal cancer (**A**) incidence and (**B**) mortality from 2020 to 2040 in Asia. The baseline scenario (represented by the black line), posits that there are no alterations in incidence and mortality, meaning that any rise in numbers is solely attributed to changes in population size and composition. Due to the unlikelihood of stable incidence rates, alternative scenarios are provided.

Among the 47 countries assessed in this study, there is an overall tendency to increase in both the incidence and the mortality rate by 2040. This projection is based on changes in the number and structure of the population, and other possible factors have not been included in the estimates. The 992,755 new cases diagnosed in 2020 in Asia are estimated to increase by 71.1%, reaching 1,698,000 new cases in 2040 (Fig. 5A, black line). In the same way, CRC mortalities in Asia are expected to increase at a rate of 85.1%, from 498,329 in 2020 to 922,000 in 2024 (Fig. 5B, black line). To ensure that fewer patients will suffer from CRC in 2040, the decreases in incidence and mortality rates must be at least 2.60% and 3.00%, respectively.

## Discussion

This is the first comprehensive analysis leveraging the updated GLOBOCAN data to evaluate the CRC prevalence, incidence, and mortality across Asia, while also examining correlations with socioeconomic development indicators and projects of future incident cases and mortalities. Our findings indicate that Asia bears a high burden of CRC in terms of incident and mortality cases, with over 2.5 million 5-year prevalent CRC cases, accounting for approximately one million new cases and 500,000 mortalities in 2020. This represents a sizable proportion of the global CRC burden, constituting 52.8% of global CRC incident cases and 54.4% of global CRC mortalities. However, the 5-year prevalence rate of CRC in Asia still remains lower than in the developed regions of the world. Our findings are mostly consistent with the previous report on Asia CRC epidemiology in 2018^8^. While there are still few reports on temporal patterns of CRC incidence in Asia, increasing incidence rates have been reported in previous studies across several Asian countries^4,22,23^, with particular increasing trends noted in young-onset CRC across the continent^24,25^. Recent evidence from Taiwan and Korea indicates substantial annual increases in early-onset CRC, with male rectal cancer rising by 3.9% in Taiwan and by 6.0% in Korea per year respectively between 1995 and 2014^24^. Globally, the age-standardized incidence rate of early-onset CRC increased from 3.05 to 3.85 per 100,000 between 1990 and 2019, with pronounced cohort effects suggesting successive generations face elevated risks^26^. A recent decomposition analysis showed East Asia alone contributed about 40% of global early-onset CRC cases in 2021, predominantly driven by population growth and birth-cohort effects^27^. These findings underscore the importance of targeted screening and preventive strategies for younger cohorts, including lifestyle interventions and potential microbiome modulation to address early-life exposures associated with CRC risk.

However, substantial heterogeneity is seen in CRC incidence and mortality across Asian subregions, with our findings suggesting Eastern Asia has 1.5 to fivefold higher ASIRs and 1.3 to fourfold higher ASMRs compared to other regions of the continent. Therefore, Eastern Asia appears as an outlier relative to the rest of the Asian regions, as both the incidence and mortality metrics mirror patterns typically observed in the more developed regions of Europe and Northern America. However, rates in the other Asian subregions still resemble the profile of less developed parts of the globe like Africa which still grapple with quite lower CRC rates.

The distinct disparity between Eastern Asia and other Asian subregions could be attributed to several factors. CRC stands for cancer with established screening methods, including non-invasive fecal occult blood tests and sigmoidoscopy or total colonoscopy^28^. At the health systems level, initiatives around public awareness, nationwide screening implementation, healthcare access, and surveillance enhancements result in more optimized case finding^29^. Accordingly, countries with stricter implementation of national screening programs and higher access to healthcare facilities are at higher odds of diagnosing CRC cases. This is also evident through our findings regarding a positive correlation between HDI and CRC incidence rates, which indicates higher incidence in more developed countries. Therefore, higher case findings in more developed Eastern Asian countries might be a contributing factor to the heterogeneities seen compared to other regions of the continent. Of particular note is Taiwan’s long-standing, organized CRC screening program, which began population-based fecal immunochemical testing (FIT) in 2004 and was later expanded to include colonoscopy for FIT-positive individuals. Using an advanced age-period-cohort-screening model, Kuo et al. demonstrated that Taiwan’s screening program reduced age-standardized CRC incidence by approximately 10% by 2019 compared with a no-screening scenario, with annual declines of 3.4% for men and 3.1% for women following program initiation^30^. Moreover, Hsiao et al. performed a population-based cohort study of over 1.5 million Taiwanese and found that consistent FIT screening was associated with a 33% reduction in CRC incidence and a 47% reduction in CRC mortality, highlighting a dose–response relationship between FIT adherence and CRC outcomes^31^. These Taiwanese data underscore the significant potential for well-implemented, organized screening programs to alter CRC trajectories and support our recommendation for prioritizing population-based screening across Asia.

On the other hand, the substantially higher rates of CRC in the East Asian population might have been driven by the increasing rate of its associated environmental and lifestyle risk factors attributable to Westernization. A recent review of gastrointestinal cancer epidemiology in Eastern Asia revealed that the overall gastrointestinal cancer incidence and mortality were higher in East Asia compared to Western regions of the world, including Northern America and Europe^32^. Notably, according to the findings of the global burden of disease study, while decreasing trends in CRC incidence were reported in Northern America over 1990–2019, CRC incidence increased rapidly in East Asia, corresponding to a 142.3% increase in ASIRs during this period^33^. These findings are mostly attributed to increasing rates of smoking, alcohol intake, and red meat diet in East Asia, which are established risk factors for gastrointestinal cancers^32,34,35^. Furthermore, genetic risk factors, such as aldehyde dehydrogenase-2 genetic polymorphisms, which are more prevalent in the East Asian population, have been linked to increased susceptibility of several gastrointestinal cancers^36^. Moreover, increased life expectancy in East Asia, as compared to other less developed Asian regions, has increased the burden of these cancers in Eastern Asia^32,37^.

Although Eastern Asian countries like Japan and South Korea bear the highest incidence rates across Asia, they possess the lowest MIR, indicating the most favorable cancer care and survival outcomes across the continent. Generally, the observed MIR in Asia (0.50) is comparable to the global average value (0.49). Notably, Eastern Asia, with a ratio of 0.49 matching the global MIR, represents the lowest MIR compared to other Asian regions. This highlights that despite Eastern Asia’s high caseload, the outcomes remain at par with international standards in terms of survival following diagnosis. Interestingly, a recent study has estimated that in the next decade, the CRC ASMR of Japan and South Korea will be surpassed by China^38^. On the other hand, while Eastern Asia mirrors worldwide MIR, South-Central has a poorer ratio of 0.60, pointing to gaps in early detection and evidence-based management in the relatively under-resourced parts of Asia. The contrasts are starker in comparison with developed regions like Northern America and Europe, with MIRs almost 25% lower than those in Asia. This possibly implies a significant number of excess mortalities in Asia that could be prevented through investments in better screening coverage, optimal treatment infrastructure, and standardization of high-quality care elements^11,39^. Reinforcing these observations, our analysis revealed a strong negative correlation between HDI and MIR values across Asia, indicating that nations with higher development levels tended to have lower MIRs or superior survival outcomes. The same correlations have been previously reported in epidemiological studies on global CRC^40^ and global gastrointestinal cancers^41^. Another recent study has also introduced a composite measure called quality of care index (QCI) for CRC, demonstrating a positive correlation between QCI and socio-demographic quintiles worldwide, which suggests that more developed countries had better CRC care^42^. Interestingly, Japan demonstrated the highest QCI among all countries. Taken together, these observations highlight that closing development gaps between regions can catalyze the quality of care disparities observed currently.

Our study findings also revealed an alarming trajectory, with Asia’s CRC burden set to escalate by over 70% in incidence and over 85% in mortality over the next decades based on anticipated population growth and aging. A previous prediction on global trends of CRC till 2035 has also predicted a rise in mortality cases by 60% and 71.5% for colon and rectal cancers, respectively^43^. Given the lack of commensurate health infrastructure expansion, resource allocation, and policy prioritization in recent times, the projected rise signals a challenge confronting Asia unless urgent strategic action is prioritized.

Despite being the first comprehensive analysis of updated GLOBOCAN data on CRC in Asia, this study has several limitations intrinsic to the GLOBOCAN methodology. The estimations rely on the most reliable country-level data; however, there persists variability in the completeness, accuracy, and representativeness of cancer registry coverage across different nations. Moreover, the anticipated projection in CRC incidence and mortality by 2040 is solely attributable to projected population growth and changes in age structure at the country level, and our analysis does not incorporate potential temporal variations in ASIRs and ASMRs within countries, which could exert an influence on future burdens.

## Conclusions

Our analysis of the latest cancer metrics reveals that CRC continues to impose a rising public health burden confronting Asia. While Eastern Asia already bears incidence and mortality rates on par with economically developed regions worldwide, lower-resourced countries across South, Southeast, and Western Asia are likely to drive growth given demographic shifts and epidemiological transitions underway. Moreover, the current cancer care and survival gaps across the continent highlight the need for improvements in underdeveloped regions of Asia. The imperative lies with health systems to refine the allocation of resources in order to enhance awareness, facilitate early detection, and deliver high-quality care.

## Supplementary Information

Supplementary Information 1.

Supplementary Information 2.

Supplementary Information 3.

Supplementary Information 4.

Supplementary Information 5.

Supplementary Information 6.

Supplementary Information 7.

Supplementary Information 8.

## Data Availability

The data used for these analyses are all publicly available at Global Cancer Observatory, United Nations Development Programme (https://hdr.undp.org/data-center/human-development-index#/indicies/HDI), and Global Health Observatory of World Health Organization [https://www.who.int/data/gho/data/indicators/indicator-details/GHO/current-health-expenditure-(che)-as-percentage-of-gross-domestic-product-(gdp)-(-)].
