# Supplementary figures and images for: Epidemiology and socioeconomic correlates of colorectal cancer in Asia in 2020 and its projection to 2040
Source: Sci Rep. 2025 Jul 22;15:26639. doi: 10.1038/s41598-025-12545-y (PMC12284187; doi:10.1038/s41598-025-12545-y)

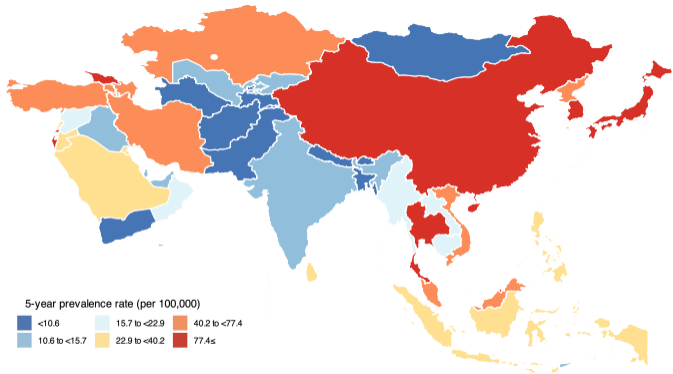

Supplement: Supplementary file 1 — Supplementary Information 1. [file 41598_2025_12545_MOESM1_ESM.pdf]

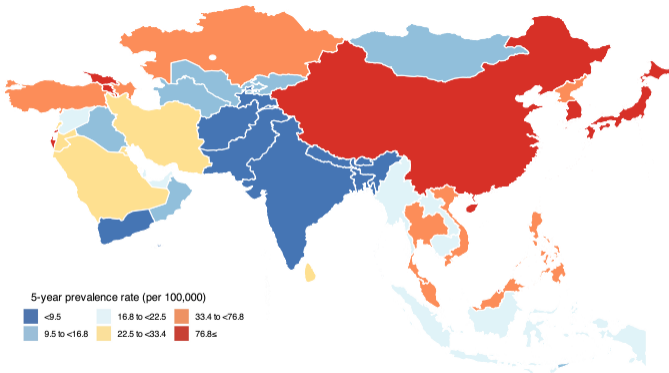

Supplement: Supplementary file 2 — Supplementary Information 2. [file 41598_2025_12545_MOESM2_ESM.pdf]

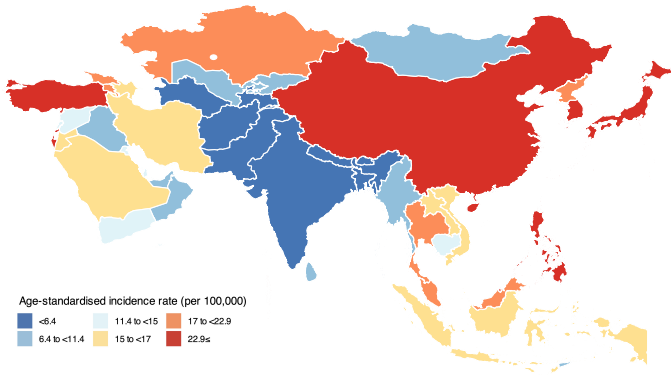

Supplement: Supplementary file 3 — Supplementary Information 3. [file 41598_2025_12545_MOESM3_ESM.pdf]

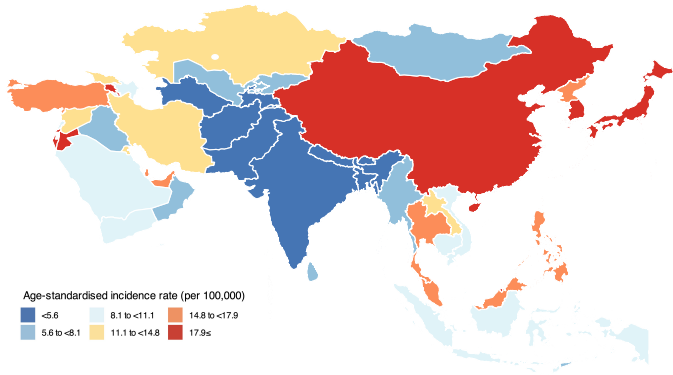

Supplement: Supplementary file 4 — Supplementary Information 4. [file 41598_2025_12545_MOESM4_ESM.pdf]

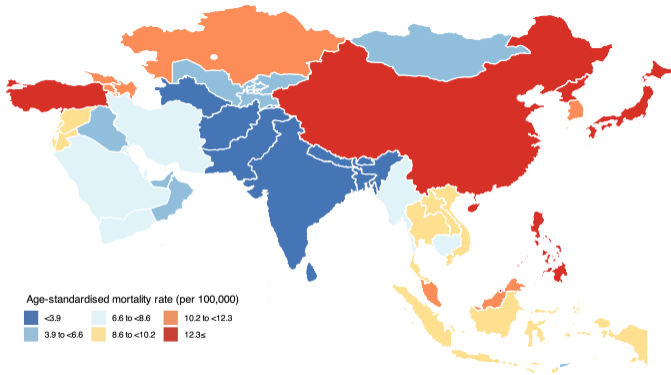

Supplement: Supplementary file 5 — Supplementary Information 5. [file 41598_2025_12545_MOESM5_ESM.pdf]

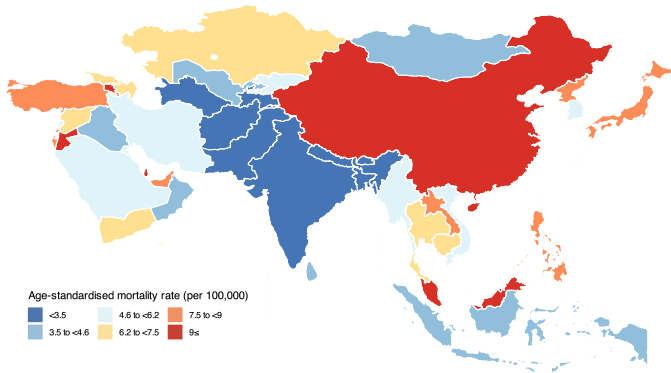

Supplement: Supplementary file 6 — Supplementary Information 6. [file 41598_2025_12545_MOESM6_ESM.pdf]
