# Supplementary material for: Epidemiology and socioeconomic correlates of colorectal cancer in Asia in 2020 and its projection to 2040
Source: Sci Rep. 2025 Jul 22;15:26639. doi: 10.1038/s41598-025-12545-y (PMC12284187; doi:10.1038/s41598-025-12545-y)
Supplement: Supplementary file 8 — Supplementary Information 8. [file 41598_2025_12545_MOESM8_ESM.docx]

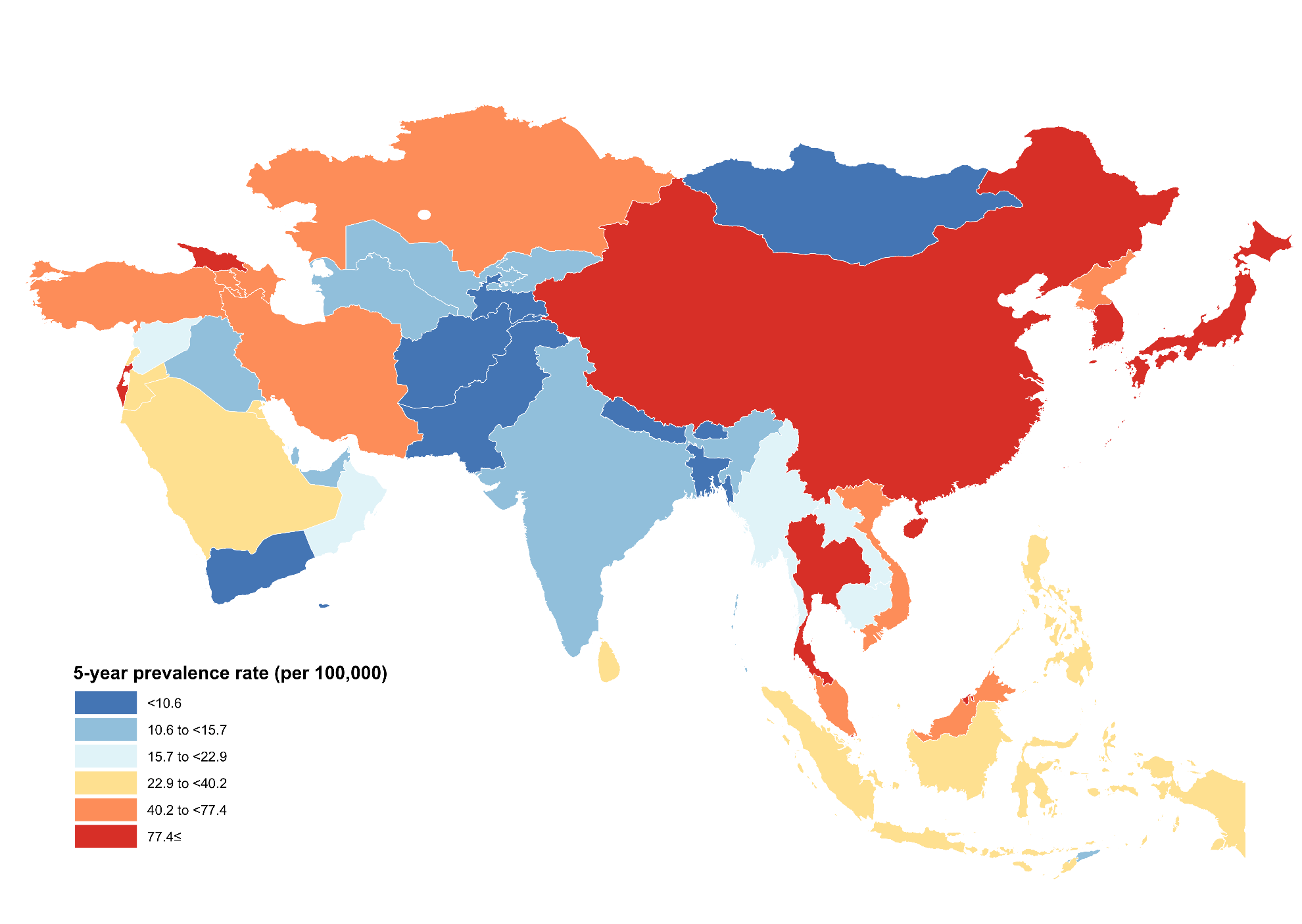


**Figure S1.** Distribution of five-year prevalence rate of male colorectal cancer in 2020 in Asia.


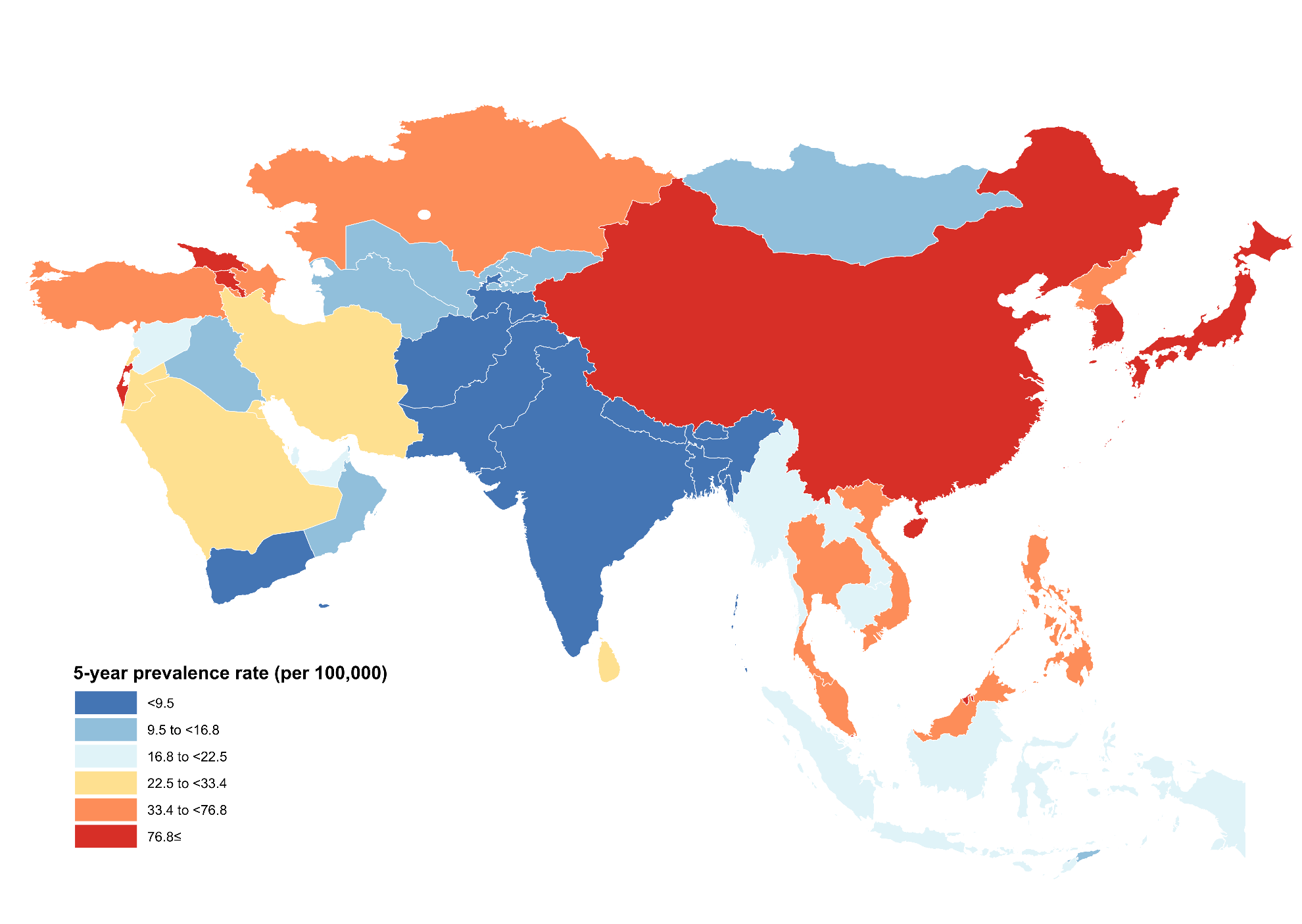


**Figure S2.** Distribution of five-year prevalence rate of female colorectal cancer in 2020 in Asia.


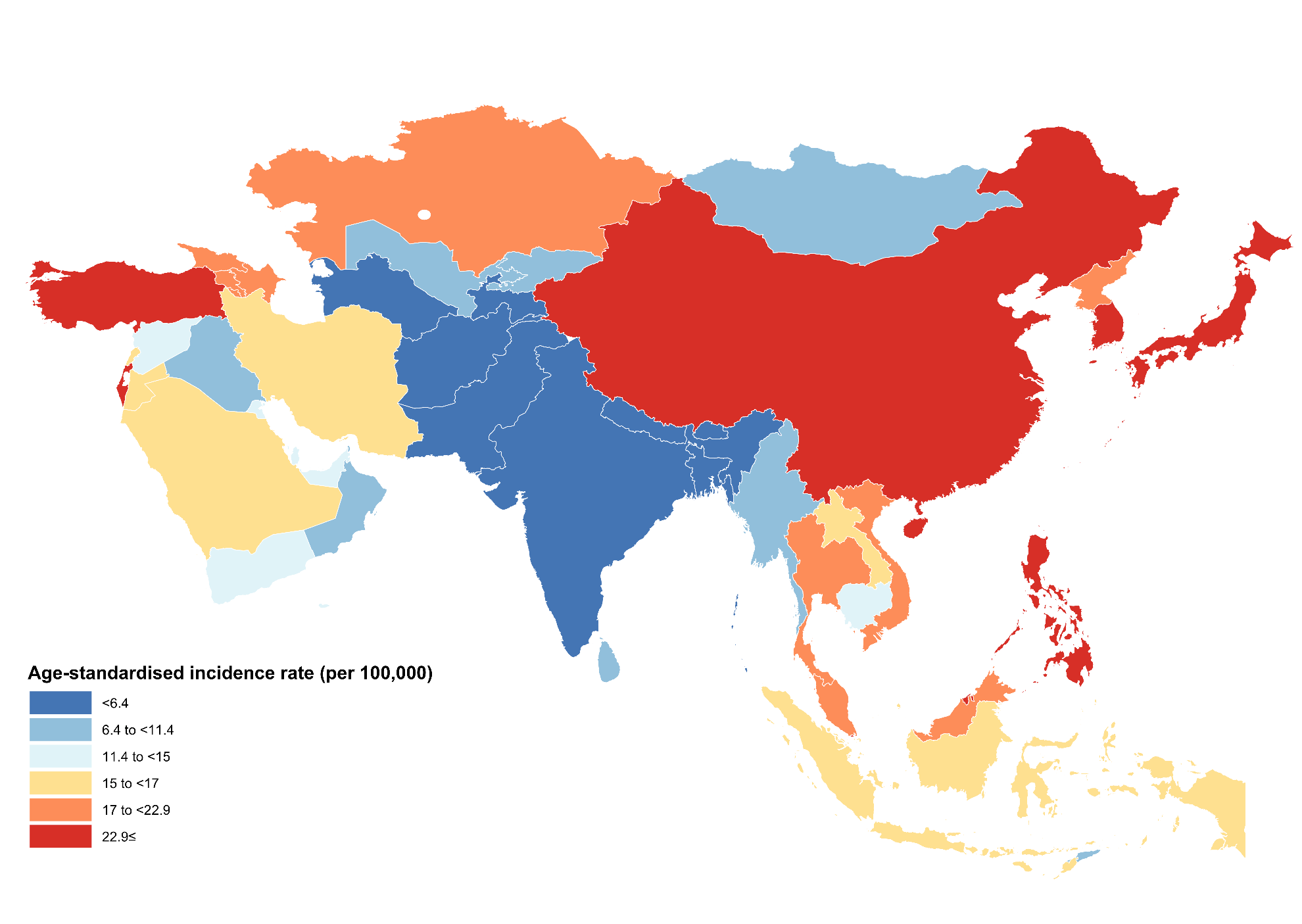


**Figure S3.** Distribution of age-standardized incidence rate of male colorectal cancer in 2020 in Asia.


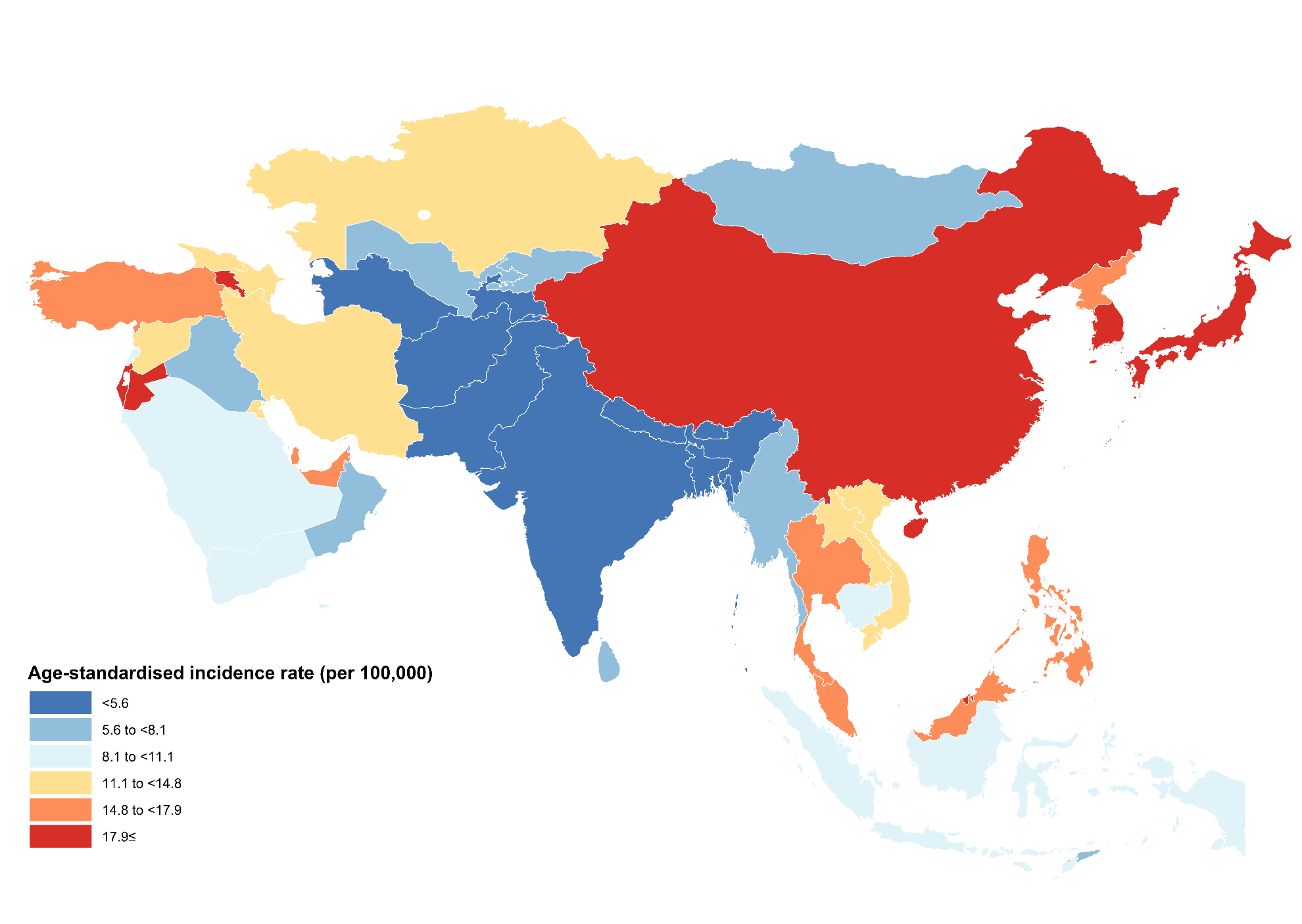


**Figure S4.** Distribution of age-standardized incidence rate of female colorectal cancer in 2020 in Asia.


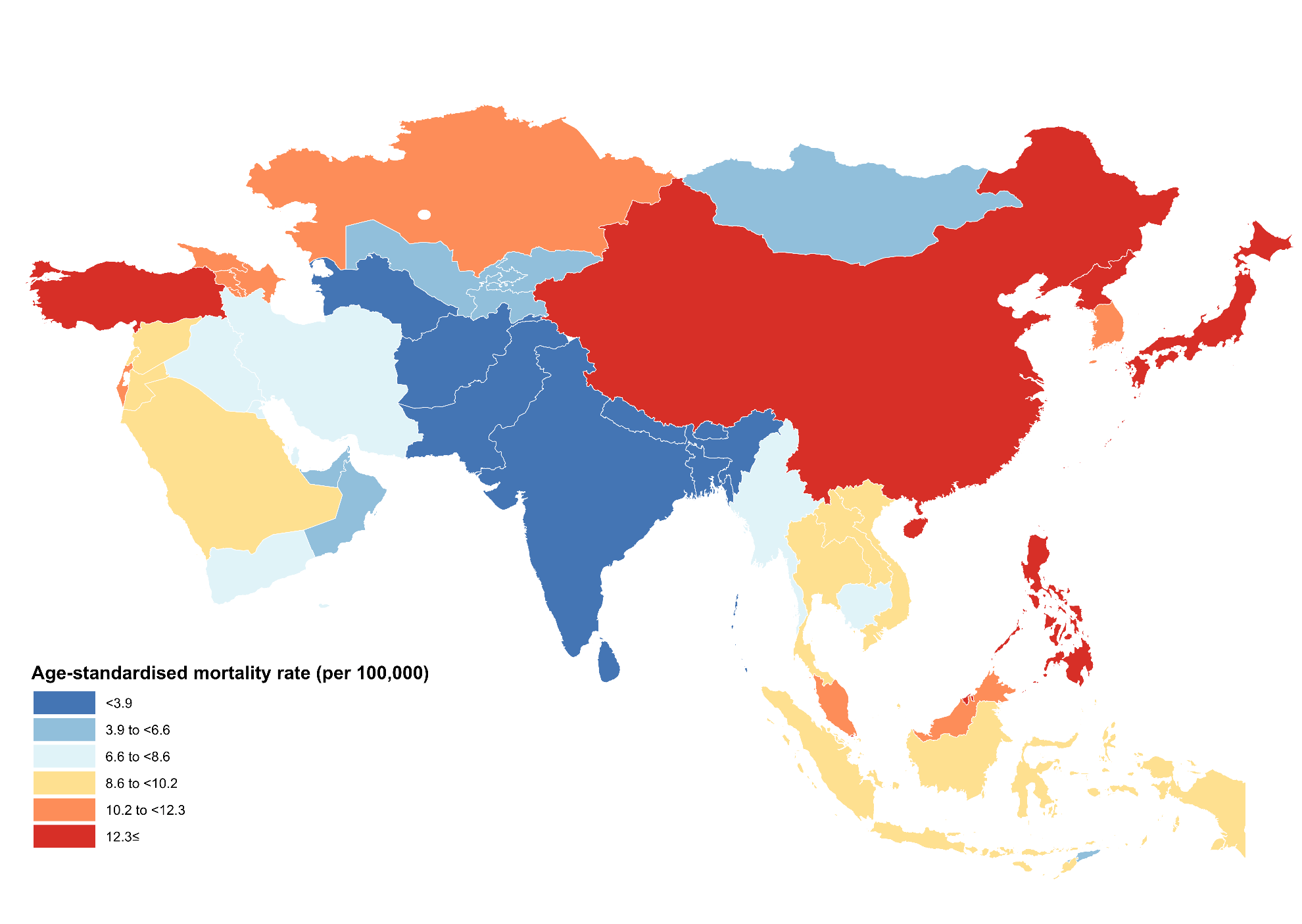


**Figure S5.** Distribution of age-standardized mortality rate of male colorectal cancer in 2020 in Asia.


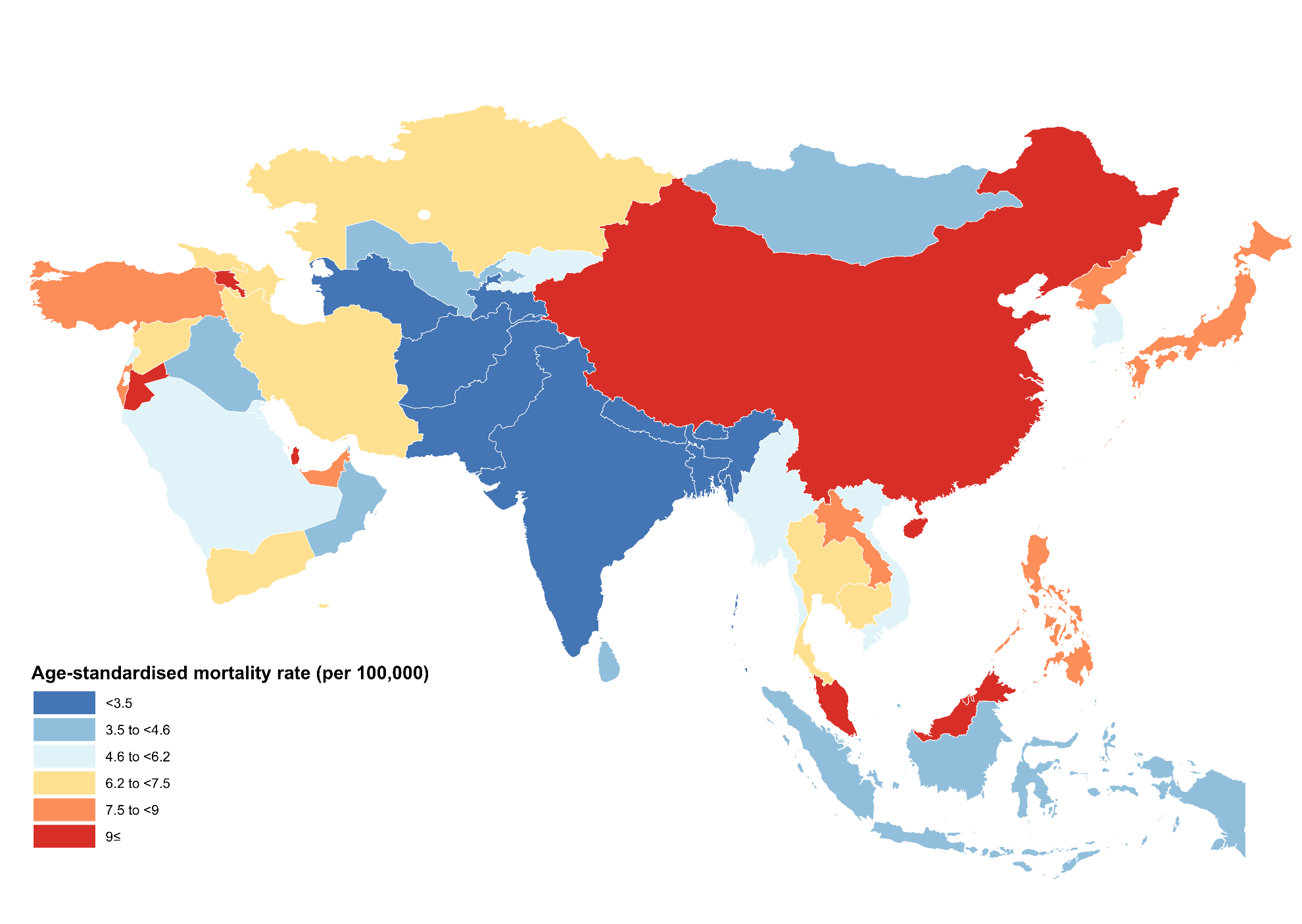


**Figure S6.** Distribution of age-standardized mortality rate of female colorectal cancer in 2020 in Asia.
